# Cardiac metastasis of a rapid-moving mixed germ cell tumour visualized by transoesophageal echocardiography

**DOI:** 10.1093/ehjcr/ytab007

**Published:** 2021-02-08

**Authors:** Toshimitsu Kato, Noriaki Takama, Tomonari Harada, Tomonobu Abe

**Affiliations:** 1 Department of Clinical Laboratory, Gunma University Hospital, Maebashi, Japan; 2 Department of Cardiovascular Medicine, Gunma University Graduate School of Medicine, Maebashi, Japan; 3 Department of Cardiovascular Surgery, Gunma University Graduate School of Medicine, Maebashi, Japan

##  

A 29-year-old man was admitted to the hospital with dyspnoea. He had a history of right testicular tumour, which was resected 1 month before admission. The pathological diagnosis was a mixed germ cell tumour. Contrast-enhanced computed tomography (CT) showed embolism in the pulmonary artery and mediastinal lymph node enlargement, indicating metastasis of the mixed germ cell tumour. No obvious contrast defects in the cardiac cavity were noted (*Panels A* and *B*). Transthoracic echocardiography (TTE) showed a mass moving rapidly between the right atrium and right ventricle (arrowhead, *Panels C* and *D*, *Video 1*). Transoesophageal echocardiography (TOE) revealed an iso-high-echoic multilocular mass extending from the superior vena cava and filling the right atrium cavity. The tip of the mass reached around the inflow of the right ventricle and was moving rapidly (arrowhead, *Panel E*, *Video 2*). Emergency tumour resection was performed (*Panel F*). The pathological diagnosis was a metastatic germ cell tumour. He underwent surgical mediastinal lymph node resection after 6 months of chemotherapy. The postoperative course was uneventful without recurrence.

**Figure:**
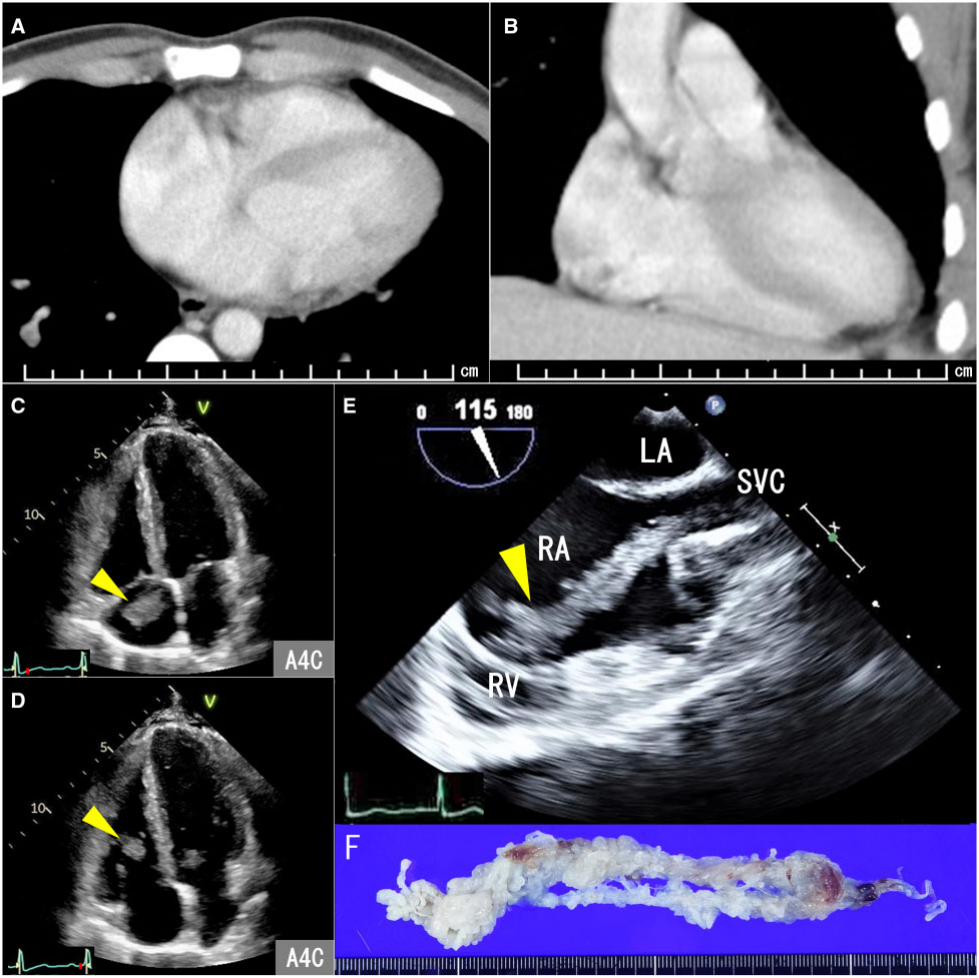


Cardiac metastases (CM) are not rare; they are encountered in 1.5–20% of autopsies of cancer patients. However, CM from testicular tumours is very rare. Computed tomography is useful for identifying CM and provides information about intra-, peri-, and extracardiac lesions. In this case, the tumour moved freely and rapidly within the cardiac cavity. Identification of such metastatic masses may be difficult on routine CT imaging. The CM was visualized on TTE, and its detailed morphology was observed on TOE. Transthoracic echocardiography and TOE are indispensable modalities to complement CT in the detection of CM, especially in an emergency case.

Ethical statement: All procedures followed were in accordance with the ethical standards of the responsible committee on human experimentation (institutional and national) and with the tenets of the Helsinki Declaration of 1964 and its later versions. Informed consent was obtained from the patient.

**Consent:** The authors confirm that written consent for submission and publication of this case report including images and associated text has been obtained from the patient in line with COPE guidance.

